# Explaining the resurgent popularity of the wild: motivations for wild plant gathering in the Biosphere Reserve Grosses Walsertal, Austria

**DOI:** 10.1186/s13002-015-0032-4

**Published:** 2015-06-30

**Authors:** Christoph Schunko, Susanne Grasser, Christian R. Vogl

**Affiliations:** Working Group Knowledge Systems and Innovation, Division of Organic Farming, Department of Sustainable Agricultural Systems, University of Natural Resources and Life Sciences (BOKU), Gregor-Mendel Straße 33, 1180 Vienna, Austria

**Keywords:** Ethnobotany, Local knowledge, Wild plant gathering, Motivations, Participatory research, Principal component analysis, Biosphere reserve, Austria, Europe, Ethnobotanik, Lokales Wissen, Wildpflanzen sammeln, Motivationen, Partizipative Forschung, Hauptkomponentenanalyse, Biosphärenreservat, Österreich, Europa

## Abstract

**Background:**

Wild plant gathering becomes again a popular and fashionable activity in Europe after gathering practices have been increasingly abandoned over the last decades. Recent ethnobotanical research documented a diversity of gathering practices from people of diverse socio-economic and cultural backgrounds who gather in urban and rural areas. Few efforts were though made to study the motivations for gathering wild plants and to understand the resurgent popularity of wild plant gathering. This paper addresses the following research questions: (1) which motivations activate wild plant gatherers? (2) which motivation-types of gatherers exist in the Grosses Walsertal? (3) how do the motivations for gathering relate to the socio-demographic background of gatherers?

**Methods:**

Field research was conducted in the Grosses Walsertal, Austria in the years 2008 and 2009 in two field research periods. Thirty-six local farmers were first interviewed with semi-structured interviews. The motivations identified in these interviews were then included in a structured questionnaire, which was used to interview 353 residents of the valley. Pupils of local schools participated in the data collection as interviewers. Principal Component Analysis was used to categorize the motivations and to identify motivation-types of wild plant gatherers. Generalized Linear Models were calculated to identify relations between motivations and the socio-demographic background of gatherers.

**Results:**

The respondents listed 13 different motivations for gathering wild plants and four motivations for not gathering. These 17 motivations were grouped in five motivation-types of wild plant gatherers, which are in decreasing importance: product quality, fun, tradition, not-gathering, income. Women, older respondents and homegardeners gather wild plants more often for fun; older respondents gather more often for maintaining traditions; non-homegardeners more frequently mention motivations for not gathering.

**Conclusions:**

The resurgent popularity of wild plant gathering comes along with an internalization of motivations: the main motivations for wild plant gathering changed from the external extrinsic motivation of gathering because of necessity towards the internalized extrinsic motivation of gathering for the highly esteemed product quality and the intrinsic motivation of gathering for the pleasure of the activity itself. This internalization of motivations supports the persistence of wild plant gathering, a positive self-perception of gatherers and good quality of engagement with wild plant gathering.

**Electronic supplementary material:**

The online version of this article (doi:10.1186/s13002-015-0032-4) contains supplementary material, which is available to authorized users.

## Zusammenfassung

**Einleitung:**

Das Sammeln von Wildpflanzen wurde in den letzten Jahrzehnten in Europa zunehmend vernachlässigt, beginnt nun aber wieder populär zu werden. Ethnobotanische Forschung berichtet von einer Vielfalt an Sammlerinnen und Sammlern, mit unterschiedlichen sozialen und kulturellen Hintergründen, die in der Stadt und am Land Wildpflanzen sammeln. Kaum eine Forschungsarbeit hat bisher aber die Motivationen der SammlerInnen genauer betrachtet und somit erforscht warum das Sammeln wieder populärer wird. Diese Arbeit zielt darauf ab 1) die Motivation für das Sammeln von Wildpflanzen im Großen Walsertal, Österreich zu ergründen und 2) und Unterschiede der Sammelmotivationen zwischen sozialen Gruppen zu beschreiben.

**Methode:**

Die Feldforschung fand in zwei Abschnitten in den Jahren 2008 und 2009 im Großen Walsertal, Österreich, statt. Im ersten Abschnitt wurden 36 Bäuerinnen und Bauern mit semi-strukturierten Fragebögen befragt. Die hier genannten Motivationen wurden danach in einem strukturierten Fragebogen aufgegriffen und mit der Unterstützung von Schülerinnen und Schülern wurden 353 BewohnerInnen mit dem Fragebogen befragt. Die Daten wurden mit Hilfe von Hauptkomponentenanalyse und Generalisierten Linearen Modellen analysiert.

**Ergebnisse:**

Die BewohnerInnen nannten 13 verschiedene Motivationen für das Sammeln von Wildpflanzen und 4 Motivationen für das Nicht-Sammeln. Diese 17 Motivationen entsprechen 5 Motivationstypen, welche in absteigender Verbreitung sind: Produktqualität, Freude am Sammeln, Tradition, Nicht-Sammeln, Einkommen. Frauen, ältere BewohnerInnen und BewohnerInnen mit Hausgarten sammeln öfter aus Freude; ältere BewohnerInnen sammeln öfter aus Tradition; BewohnerInnen die keinen Hausgarten bewirtschaften nennen öfter Gründe nicht zu sammeln.

**Schlussfolgerungen:**

Die rückkehrende Popularität des Sammelns von Wildpflanzen geht mit einer Internalisierung der Motivationen einher: Wildpflanzen werden heute nicht mehr aus Notwendigkeit gesammelt (externe extrinsische Motivation) sondern vor allem wegen deren guten Produktqualität (internalisierte extrinsische Motivation) und aus Freude an der Tätigkeit (intrinsische Motivation). Diese Internalisierung der Motivationen fördert die Beständigkeit des Wildpflanzensammelns, eine positive Selbstwahrnehmung der SammlerInnen und eine gute Qualität des Sammelguts.

## Background

Wild plant gathering becomes again a popular and fashionable activity in Europe after gathering practices have been increasingly abandoned over the last decades. Ethnobotanical research documented a diversity of gathering practices and people of diverse socio-economic and cultural backgrounds gather in urban and rural areas [1, 2].

Local knowledge about wild plant gathering historically was transmitted from generation to generation. Such traditional mechanisms were neglected in the last decades due to the decreasing necessity of wild plant gathering for subsistence. These days wild plant knowledge also gets transmitted via scientific and popular publications in print and online media, in books and field guides, through excursions, agrotourism, field courses, internet databases, avant-garde cuisine and food and health related associations [1]. These reports may pick up or renew traditional practices, mingle traditional with modern practices or report about new practices and most often are decontextualized and do not refer to sources of origin [3].

The plants gathered in Europe belong to diverse plant families and plant species. In general most people gather occasionally and a small diversity of plant species and only few people gather frequently and a diversity of different species [4]. Most wild plants are gathered and used for food and medicine. The people most knowledgeable about wild plants are frequently elderly persons and women [2, 5].

Traditional and modern wild plant practices were documented and observed in many regions all over Europe. However, few efforts were made to study in detail why people gather wild plants these days, when its necessity widely faded. The motivations encountered include health-conscious nutrition, poor economy and generating income for the poor and the elderly in the case of wild food plant gathering in Hungary [6], supporting subsistence and generating income in Bulgaria [7], spending a pastime and generating income for immigrants and foreign seasonal labor, especially in urban regions in Sweden [8] and bringing joy [9, 10], obtaining healthy and high quality products, ensuring subsistence, maintaining traditions, saving money and gaining income in Austria [10].

Although this versatile array of motivations was indicated for wild plant gathering, few studies systematically analyzed the motivations for wild plant gathering in a given region in Europe (notable exception: [10] who included questions concerning the various motivations for wild plant gathering in semi-structured interviews with 16 respondents). Understanding motivation is though essential for explaining and predicting human behavior [11] and therefore essential for understanding why plants are gathered in Europe today.

We adopt the definition of Ryan and Deci, who describe motivation as a continuum ranging from “amotivation or unwillingness, to passive compliance, to active personal commitment”, and define that: “To be motivated means to be moved to do something. A person who feels no impetus or inspiration to act is thus characterized as unmotivated, whereas someone who is energized or activated toward an end is considered motivated.” Ryan and Deci further distinguish between intrinsic and extrinsic motivations, the first referring to “doing something because it is inherently interesting or enjoyable” and the later referring to “doing something because it leads to a separable outcome” [12].

The more intrinsic and therefore internalized a motivation is, the greater the persistence, the more positive the self-perception and the better the quality of engagement in an activity [12]. Intrinsic motivation guarantees a sizable commitment with an activity. Extrinsic motivation in contrast includes various degrees and qualities, from fully externally driven activities to self-endorsed extrinsic motivation [12]. Also here, the more internalized and therefore self-endorsed an extrinsic motivation is, the better the quality of engagement in an activity. In the context of wild plant gathering, the intrinsic motivation would be gathering because it is inherently enjoyable while an example for a fully externally driven motivation would be to gather to cover the daily nutrition needs or to gain income, both separable outcomes.

This paper aims 1) to identify the motivations for wild plant gathering in the Grosses Walsertal Biosphere Reserve, Austria and 2) to describe the links between motivations and the socio-demographic background of gatherers.

The paper addresses the following research questions: (1) which motivations activate wild plant gatherers? (2) which motivation-types of gatherers exist in the Grosses Walsertal? (3) how do the motivations for gathering relate to the socio-demographic background of gatherers?

## Methods

### Field site

Field research was conducted in the Grosses Walsertal Biosphere Reserve, Austria in the years 2008 and 2009 in two field research periods.

**Fig. 1:**
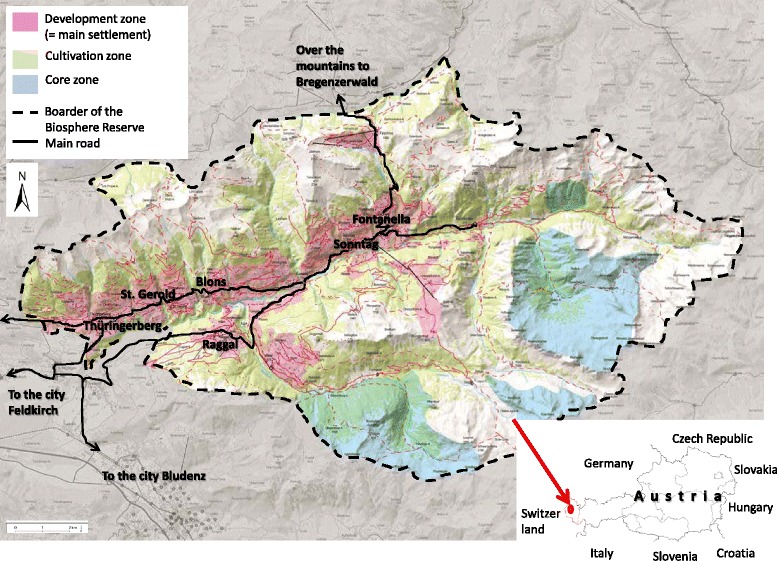
Map of the Biosphere Reserve Grosses Walsertal (Source: [13], modified)

The Grosses Walsertal (GWT) is a mountain valley characterized by alpine farming and is situated in Vorarlberg, the very western province of Austria (Fig. 1). Approximately 3.400 people live there in an area of 192 km^2^. The remote location of the region supported the creation and conservation of a distinct culture including a specific dialect, the *Walserdeutsch*. The valley is shaped by meadows and pastures due to long-lasting livestock husbandry [13]. There are 180 active farmsteads in the valley of which 40 % are run organically. About 37 % of the inhabitants of the GWT work in the GWT–16 % in small trade enterprises, 11 % in agriculture, 8 % in tourism, and 3 % in public service–whereas 61 % commute outwards the valley for work [14]. Since the year 2000, the GWT has been acknowledged as UNESCO Biosphere Reserve [13] (see our publications for more information on the field research area [9, 15].

### Data collection and sampling

Between July and September 2008, 36 local farmers (34 women, 2 men) were interviewed about wild plant gathering using freelists and semi-structured interviews [16]. Field notes were taken during the interviews to record the information given and brain protocols were completed afterwards. The interviews were also recorded using a digital voice recorder. The motivations were mentioned by the interviewees spontaneously during the course of the interviews and were not elicited in distinct questions about motivations. The exact wording of the motivations was composed by the second author based on the field notes and brain protocols.

The motivations were then included in a structured questionnaire in the second field research period in spring of the year 2009 and informants were asked to rate the motivations given. The questionnaire was structured as a table and was pretested with two interviewees. The motivations were listed in the rows and the respondents were asked to mark in the columns if they apply to their personal motivations for collecting wild plants using Likert scales with the values one (full accordance) to five (no accordance). In the questionnaire the respondents were also asked to state their gender, age and if they work in a homegarden.

Data was collected in a participatory way, with the support of pupils as interviewers. The second author organized wild plant workshops in the seven primary schools of the valley to prepare the pupils, aged six to ten, for the topic of wild plant gathering. At the end of these workshops she presented the questionnaire and subsequently asked the pupils to fill in the questionnaire with several family members separately as homework. Hence, the pupils represented the interviewers in this study (like done by e.g. [7] before). Every pupil received four copies of the questionnaire. The teachers were asked to collect the questionnaires, once filled in. This sampling strategy allowed us to gather information from a large number of people living in the GWT. However, it also created some bias since people without a connection to children aged six to ten were not reached.

### Data analysis

**Fig. 2:**
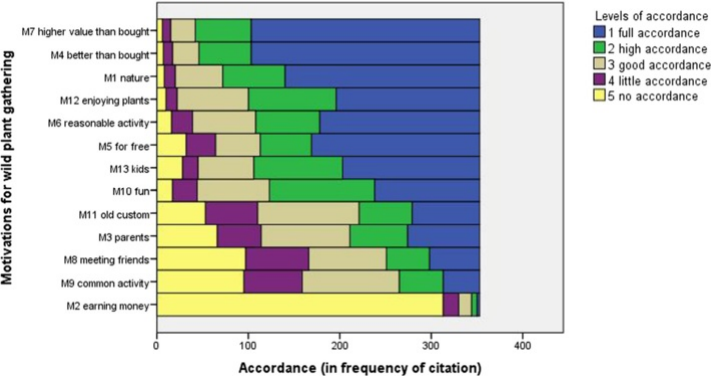
Importance of the thirteen motivations for wild plant gathering in the Grosses Walsertal, *n* = 353

Data was first analyzed by frequencies and percentages (see Additional file 1: Table S1 for raw data). Principal component analysis (PCA) using Varimax rotation was then completed to group the 17 motivations into smaller sets of answer themes. The effects of the socio-demographic variables sex (male/female), age (in years) and homegardening (yes/no) on the affiliation with the identified components was then calculated using Generalized Linear Models (GLM). The regression based factor scores were thereby used as depending variable. For creating the GLM, we chose the linear model type, included only main effects, selected Type III analyses, Wald statistics as well as the usual significance level of *p* = 0.05 for identifying significant relations. Missing values were treated as missing listwise in the calculations. All statistical calculations and the design of Fig. 2 were completed in SPSS 21 [17].

In total, 506 questionnaires were returned by 189 pupils. Hence about 15% of the population of the valley was reached with the survey. In 353 questionnaires the motivations for wild plant gathering were filled in completely. These questionnaires were used for descriptive statistics and PCA. However, ten of these respondents did not indicate their age and the answers of 343 respondents were used in the subsequent GLM.

In GLM, the regression coefficient B gives information about the strength of the relation and can be directly interpreted. For example in the case of gender, if B = 2, women listed on average two plant species more than men in the respective use category; in the case of age, if B = 0.1, respondents listed on average 0.1 plant species more with every additional year of age.

The sample consisted of 108 male and 245 female individuals ranging from 8 to 83 years of age (median: 42 years). Two-hundred-twenty-two of the informants are homegardeners, 130 do not homegarden.

### Ethical considerations

We followed the Code of Ethics of the International Society of Ethnobiology in our research activities [18]. In the parts where the research involved children, we followed the *International Charter for Ethical Research Involving Children* [19]. In the following, we draw on the seven key commitments of the Charter for discussing the ethical conduct of our study in relation to the involvement of children.

We followed commitment one “Ethics in research involving children is everyone’s responsibility” and two “Respecting the dignity of children is core to ethical research” in planning the research process and in all our interactions with children.

We followed commitment 3 “Research involving children must be just and equitable” by ensuring that all project related tasks were co-designed with teachers, adapted to the knowledge level of pupils and included in the school routine. It was ensured that the project related tasks did not provide extra-labor to the pupils.

We followed commitment 4 “Ethical research benefits children” through maximizing the learning experience of the pupils. The second author organized wild plant-workshops with the pupils before and after the data collection to pre-inform about the topic and return results respectively. The questionnaire was designed in a child-oriented way and provided opportunities to learn about wild plants. Parts of the results of the study were published in the local newsletter of the Biosphere Reserve.

We followed commitment 5 “Children should never be harmed by their participation in research” through being attentive during all interactions with the pupils and avoiding any potential risks of harm when planning the study. We especially ensured that the workload for pupils remained balanced and no pressure of completing the homework is exerted.

We followed commitment 6 “Research must always obtain children’s informed and ongoing consent”. We obtained prior informed consent from the Biosphere Reserve committee, the school directors, commitment of the committee for education and culture, and the parents of the involved children. The project activities were also pre-announced in the local newsletter, received by every household of the valley. Informed assent from the pupils was sought during the first workshops. The children were then informed about the study and given the opportunity to dissent. However, we are aware that in school settings children may easily feel obliged to co-operate [20]. We did not receive any objections to participate in the research activity.

We followed commitment 7 “Ethical research requires ongoing reflection” in all interactions with the children through reflecting upon our practices and values and their influence on the pupils.

### Remarks and limitations of the study design

The motivations used for the structured interviews were almost exclusively proposed by female farmers, identified through snowball sampling. Hence, as we did not use a stratified sample, we cannot be sure that the motivations found contain all motivations relevant for wild plant gathering in the GWT.

Also, the motivations used were listed by the interviewees spontaneously during the course of the interviews and were not elicited in distinct questions about motivations. Including such distinct questions might have resulted in further motivations.

We took several precautions to ensure good data quality and responsiveness of informants. These included developing the questionnaire together with teachers and local actors and in a child-oriented way, two pre-tests in real interview settings, the conduct of preparation workshops for the children taking part in the study, pre-information of the local population about the study by means of a local newsletter, an information letter for the parents of the participating children and an information meeting with some parents. However, as is the case with many other methods where the investigator does not directly witness the collection of data, the children and their informants filled in the questionnaires without the attendance of a researcher and we therefore cannot guarantee that there were no misunderstandings in answering the questionnaires and that each questionnaire was really filled in by one person at a time and without being influenced.

## Results

### Descriptive statistics

The farmers mentioned 13 motivations for gathering wild plants and four motivations for not gathering in the semi-structured interviews (Table 1).

**Table 1:** Motivations for (not) gathering wild plants in the Biosphere Reserve Grosses Walsertal, in German and English translation

Motivation Number	Motivations for gathering wild plants…	…in German	… in English translation
1	Ich sammle wildwachsende Pflanzen, weil… / I gather wild plants because…	…ich gerne in der Natur draußen bin	…I like to be in nature
2		…ich damit etwas Geld verdiene	…I earn some money
3		…das die Mutter/der Vater immer schon gemacht haben	…my mother/father always used to do that
4		…selbst Gesammeltes/selbst Gemachtes besser ist als gekaufte Produkte	…self-made products are better than bought ones
5		…es nichts kostet	…they are for free
6		…es eine sinnvolle Beschäftigung in der Freizeit ist	…it is a reasonable engagement in the leisure time
7		…Produkte aus selbstgesammelten Pflanzen einen größeren Wert haben für mich als gekaufte Produkten	…products made from self-gathered plants have a higher value for me than bought products
8		…ich dabei Freunde treffe	…I meet friends
9		…das im Großen Walsertal so üblich ist	…people in the Grosses Walsertal use to do that
10		…das Sammeln Spaß macht	…gathering is fun
11		…es ein alter Brauch ist	…it is an old custom
12		..ich an den Pflanzen Freude habe	…I like plants
13		…ich mit den Kindern/Enkelkindern gemeinsam draußen etwas machen kann	…I can do something together with my kids/grandchildren
14	Und wenn ich keine wildwachsenden Pflanzen sammle, dann ist das weil… / And when I do not gather wild plants, this is because…	…ich keine dafür Zeit habe	…I do not have time
15		…man‘s eh kaufen kann	…I can buy them
16		…ich mich mit Pflanzen nicht gut genug auskenne	…I do not know plants well enough
17		…es mir zu viel Aufwand ist	…it’s too much work

The analysis of the structured questionnaires shows that the most frequently cited motivations for gathering wild plants in the Grosses Walsertal (GWT) are the perceived higher value of wild gathered plants compared to bought ones (88.1 % of the respondents have full accordance or high accordance with this reason), that self-made products are perceived as better than bought ones (86.9 %) and that people enjoy being in nature when gathering (79.6 %). Earning money with selling wild plants is the least mentioned motivation for wild plants gathering (2.5 %). The most important motivation for not gathering is the lack of time (43.6 %) (Table 2, Fig. 2).

**Table 2:** Answer frequencies (freq) and percentages (%) for motivations for gathering wild plants (*n* = 353), Likert scale ranging from 1 (full accordance) to 5 (no accordance)

Motivations for gathering wild plants		1	2	3	4	5	Total
(1) Nature	freq	213	68	52	12	8	353
	%	60.3	19.3	14.7	3.4	2.3	100
(2) Earning money	freq	3	6	14	17	313	353
	%	0.8	1.7	4.0	4.8	88.7	100
(3) Parents	freq	79	63	97	48	66	353
	%	22.4	17.8	27.5	13.6	18.7	100
(4) Better than bought	freq	250	57	29	10	7	353
	%	70.8	16.1	8.2	2.8	2.0	100
(5) For free	freq	184	56	49	32	32	353
	%	52.1	15.9	13.9	9.1	9.1	100
(6) Reasonable activity	freq	175	70	69	23	16	353
	%	49.6	19.8	19.5	6.5	4.5	100
(7) Higher value than bought	freq	250	61	27	9	6	353
	%	70.8	17.3	7.6	2.5	1.7	100
(8) Meeting friends	freq	55	47	85	69	97	353
	%	15.6	13.3	24.1	19.5	27.5	100
(9) Common activity	freq	40	48	106	64	95	353
	%	11.3	13.6	30.0	18.1	26.9	100
(10) Fun	freq	115	115	79	27	17	353
	%	32.6	32.6	22.4	7.6	4.8	100
(11) Old custom	freq	74	58	111	57	53	353
	%	21.0	16.4	31.4	16.1	15.0	100
(12) Enjoying plants	freq	157	96	78	12	10	353
	%	44.5	27.2	22.1	3.4	2.8	100
(13) Kids	freq	150	97	61	17	28	353
	%	42.5	27.5	17.3	4.8	7.9	100
Motivations for not gathering wild plants		1	2	3	4	5	Total
(14) No time	freq	85	68	79	45	76	353
	%	24.1	19.3	22.4	12.7	21.5	100
(15) Prefer to buy	freq	20	31	87	65	150	353
	%	5.7	8.8	24.6	18.4	42.5	100
(16) Lack of knowledge	freq	45	87	107	64	50	353
	%	12.7	24.6	30.3	18.1	14.2	100
(17) Much Work	freq	25	50	102	75	101	353
	%	7.1	14.2	28.9	21.2	28.6	100

### Principal component analysis

Principal component analysis allocates the 17 motivations for gathering and not gathering wild plants into five components with eigenvalues higher than 1. The five components explain 59.9 % of the variance (Table 3). The Kaiser-Meyer-Olkin measure indicated the adequacy of the data for PCA (KMO = 0.840). Bartlett's test of sphericity indicated that correlations between items were sufficient for PCA (*p* < 0.001).

**Table 3:** Variance explained in principal component analysis through extracted factors and eigenvalues (*n* = 353)

Component	Initial eigenvalues		
	Total	% of variance	Cumulative %
1	4.736	27.862	27.862
2	2.187	12.864	40.725
3	1.183	6.959	47.685
4	1.070	6.294	53.978
5	1.014	5.966	59.944
6	0.862	5.071	65.015
7	0.769	4.522	69.537
8	0.715	4.204	73.741
9	0.630	3.704	77.445
10	0.618	3.634	81.079
11	0.601	3.537	84.616
12	0.547	3.215	87.831
13	0.536	3.154	90.985
14	0.444	2.612	93.597
15	0.407	2.393	95.989
16	0.392	2.306	98.295
17	0.290	1.705	100.000

Component one accounted for 27.9 % of variance and comprised five items. These items reflect fun as a principal motivation for wild plant gathering. Gathering wild plants because of fun is widespread among the respondents and on average 71.2 % of the respondents has full accordance or high accordance with the five motivations of this component. Component two accounted for 12.9 % of variance and comprised four items. These items reflect tradition as a motivation and on average 32.9 % of the respondents has full accordance or high accordance with the four motivations. Component three accounted for 7.0 % of variance and comprised four items. This component comprises the motivations for not gathering wild plants and on average 29.1 % of the respondents has full accordance or high accordance with these four motivations. Component four accounted for 6.3 % of variance and comprised three items which reflect a quality motivation. This type of wild plant gatherer is the most widespread and on average 81 % had full accordance or high accordance with the three motivations. Component five accounted for 6.0 % of variance and comprised one item, which reflects the motivation of generating income. This motivation was the least one mentioned and only 2.5 % had full accordance or high accordance with this motivation (Table 4).

**Table 4:** Factor loadings from principal component analysis on seventeen motivations for gathering wild plants (*n* = 353)

Motivations for (not) gathering wild plants	Factor loadings				
	1	2	3	4	5
(10) Fun	,778				
(13) Kids	,700				
(12) Enjoying Plants	,641				
(6) Reasonable activity	,635			,400	
(1) Nature	,607				
(9) Common activity		,812			
(11) Old custom		,776			
(8) Meeting friends		,630			
(3) Parents		,508			
(17) Work			,767		
(16) Lack of knowledge			,720		
(15) Buy			,661		
(14) No time			,534		,436
(4) Better than bought				,771	
(7) Higher value than bought				,705	
(5) For free				,639	
(2) Earning money					,906

### Generalized linear models

Five of the fifteen relations resulted significant between the variables sex, age and homegardening and the factor scores of the five extracted components in PCA (Table 5).

**Table 5:** Generalized Linear Models showing effects of sex, age and homegardening on regression based factor scores of components from principal component analysis (*n* = 343)

Regression based factor scores	Sex		Age		Homegardening	
	(male, female)		(in years)		(yes, no)	
	Regr B^a^	*p*	Regr B	*p*	Regr B	*p*
Factor 1–Fun	0.341	0.02*	−0.010	0.01*	−0.399	0.000*
Factor 2–Tradition	−0.074	0.515	−0.012	0.000*	0.030	0.787
Factor 3–Not gathering	−0.067	0.566	−0.001	0.859	0.254	0.028*
Factor 4–Product Quality	0.139	0.228	−0.001	0.765	−0.207	0.070
Factor 5–Income	−0.017	0.884	0.004	0.217	0.001	0.993

^a^ Regression Coefficient B

* significant at 0.05 significance level

Women (Regr B = 0.341; *p* = 0.02), older respondents (Regr B = −0.010; *p* = 0.01) and homegardeners (Regr B = −0.399; *p* = 0.000) gather wild plants more often for fun than men, younger respondents and non-homegardeners. Older respondents gather more often for maintaining traditions than younger respondents (Regr B = −0.012; *p* = 0.000). Non-homegardeners more frequently mention motivations for not gathering compared to homegardeners (Regr B = 0.254; *p* = 0.028).

## Discussion

Product quality and fun are the principal motivations for wild plant gathering and for most people in the Grosses Walsertal (GWT) both, and not only one of them, apply. Gathering for maintaining tradition was mentioned less frequently and gathering for income is done by a small number of people only. Earlier research identified similar types of wild plant gatherers such as commercial (here income), recreational (here fun) or subsistence-based gathering (here included in product quality) [21]. These motivation-types are discussed separately below as well as the not gatherer-type.

### Product quality-type

**Fig. 3:**
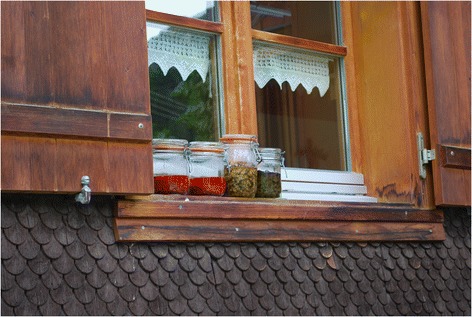
Wild plant products mature in the sun. Obtaining self-made high quality products is a principal motivation for wild plant gathering (Photo: S. Grasser)

The product quality type of wild plant gatherer is guided by the extrinsic motivation of obtaining self-made high quality products at low cost. The product, a separable outcome and thereby a characteristic for an extrinsic motivation, is thus central for this type (Fig. 3).

This type is most widespread but has little explanatory power in the Principal Component Analysis (this component accounted for only 6.3 % of variance) and is not linked to the socio-demographic variables investigated. Product quality is therefore a widely and equally distributed motivation and similarly pronounced across people disregarding their sex, age and homegardening practices.

Quality is a general term comprising versatile meanings. Quality criteria include taste, color, consistency, processing properties for conservation or preparation, pharmacological effectiveness, naturalness, purity and others. Without knowing precisely how wild plant quality is understood by the respondents of this study, we understand that from an emic perception wild plant products have favorable properties in general and especially when they are homemade and free to harvest.

In Eastern Tyrol, situated in the western Alps of Austria, quality is also a main motivation for gathering wild plants. There, 63 % of the respondents get motivation to gather wild plants because of health reasons, which are inherently linked to high product quality, and 38 % by the natural and pure quality of homemade products derived from wild plants [10].

The high quality of wild plants is supported in general terms by nutrition science, but there is a lack of systematic research and databases of nutritional compounds and health properties of wild plants for detailed analyses [22–24]. Research so far highlighted the value of wild food plants as functional foods [25, 26], the central role of wild plants in selected diets and associated health benefits [27, 28], the important amounts of bioactive compounds present [2, 29] and the extraordinary antioxidant properties of herbaceous wild plants [30, 31]. Additionally, wild food plants enhance the diversity of foods eaten and contribute to a wholesome diet, rich in diverse nutrients [32].

However, wild food plants are also increasingly exposed to pollution and changes in agro-ecological systems [1]. Pollution is however less an issue in the Grosses Walsertal since it is recognized as a Biosphere Reserve and local people emphasize their close relationship with the pollution-free environment [9, 13]. Living in this environment may have enhanced the pronounced perception of wild plants as bearing high quality. Research on the motivations for wild plant gathering in other regions will show if the product quality type of gatherers is similar pronounced elsewhere. Especially research in areas exposed to more pollution, such as semi-urban or urban areas, might find different results.

Ethnobotanical research on motivations for homegardening in three Spanish regions highlighted quality as a major, but unevenly pronounced, motivation. In Central Asturia 12 % of respondents, in the Sierra Norte de Madrid 36 % and in the Catalan Pyrenees 58 % of respondents get motivation for homegardening because of quality reasons [33]. In Eastern Tyrol, Austria, the main motivation for homegardening, cited by 76 % of respondents, is to obtain homegrown food (including certainty about the origin of food and production methods) [34], which is part of the product quality in the Grosses Walsertal. These comparable results for homegardening suggest that high quality products may be a pervasive motivation for several ethnobotanical activities.

### Fun-type

Fun is the second major motivation for gathering wild plants in the GWT and the most extensive component with most explanatory power in the Principal Component Analysis.

**Fig. 4:**
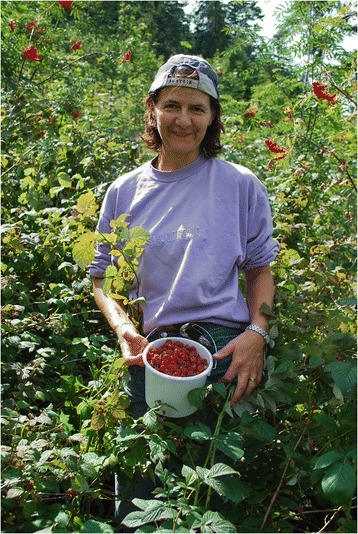
Picking of raspberries from wild populations. Fun is the second major motivation for gathering wild plants (Photo: S. Grasser)

**Fig. 5:**
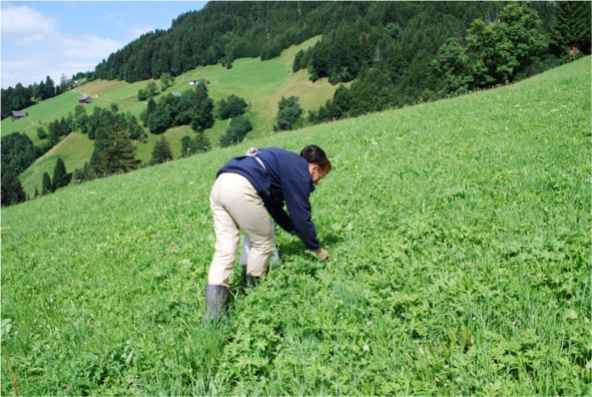
Gathering wild herbs in alpine scenery. Being outside and in interaction with nature while gathering contributes to the fun of the activity (Photo: S. Grasser)

Wild plant gathering is perceived as enjoyable for this type of wild plant gatherers. These gatherers enjoy being outside, interacting with nature and plants and bringing kids along. They gather wild plants out of pleasure and experience it as a time-out in a calm environment (Figs. 4 and 5). This motivation changed from wild plant gathering in the past when it was a necessity because of poverty and when the related widespread motivation was fulfilling the daily needs [9].

The society in the GWT has, over the last decades, quickly transformed from a society based on agriculture to a society of workers and employees. Wild plant gathering was thereby not only abandoned, like found in other regions as well [2], but also revitalised [9]. Fun replacing necessity as a motivation indicates that an internalisation process took place, hence the motivation for wild plant gathering shifted from the extrinsic motivation of gathering for obtaining wild plant products to the intrinsic motivation of gathering because of the activity itself. Now gathering wild plants is perceived as a joyful activity and hence bears the characteristics of an intrinsic motivation along with its positive connotations of greater persistence, more positive self-perception and better quality of engagement compared to extrinsic motivations [12].

In Eastern Tyrol, fun was stated as a major reason for gathering wild plants as well, mentioned by 75 % of the respondents [10].

Women, older respondents and homegardeners gather wild plants more often for fun than men, younger respondents and non-homegardeners and homegardeners less frequently do not gather wild plants in the GWT.

Women and older respondents were frequently identified as principal gatherers of wild plants [2, 5] and they are not only the principal gatherers in the GWT as well but also enjoy this activity.

Homegarden research in three Spanish regions elicited similar results like in the GWT and enjoying the activity was even the most frequently mentioned motivation there [33]. This indicates that also fun, along with product quality, is a pervasive motivation for several ethnobotanical activities.

The fieldwork for the project at hand has also directly shown the capacity for fun and enthusiasm for wild plant gathering in the GWT. School children, parents and elders were involved in the research process and these processes and the resulting participatory films, books and brochures illustrate the enthusiasm for wild plant gathering in the GWT [9, 35–37].

### Tradition-type

**Fig. 6:**
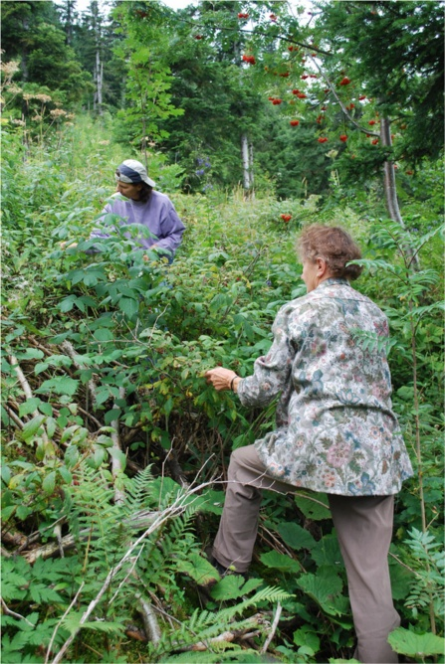
Mother-in-law and daughter-in-law pick raspberries together in the forest. Intergenerational learning and carrying on customs motivate to gather wild plants (Photo: S. Grasser)

The traditional gatherer type derives motivation for wild plant gathering from carrying on activities learned from their parents. This type perceives plant gathering as an old custom and a common activity. Meeting friends is connected with this gatherer-type, which indicates that wild plant gathering traditionally was linked with group activities (Fig. 6).

There is accordance that traditional practices of wild plant gathering are on decline in many regions of Europe and maintained mainly by elderly persons [1, 2]. This overlaps with our results; in the GWT older respondents gather more often for maintaining traditions than younger respondents. However, as shown above, in the GWT younger generations find alternative motivations for wild plant gathering and especially product-quality is a widespread motivation of all generations and people with diverse backgrounds. This adds to the claim that traditional practices are abandoned and new practices emerge [1] and shows that also the motivation for gathering changes in line with changing practices.

In Eastern Tyrol, 13 % of the respondents mentioned maintaining traditions as a reason for gathering wild plants [10], compared to 32.9 % in this research.

### Income oriented-type

**Fig. 7:**
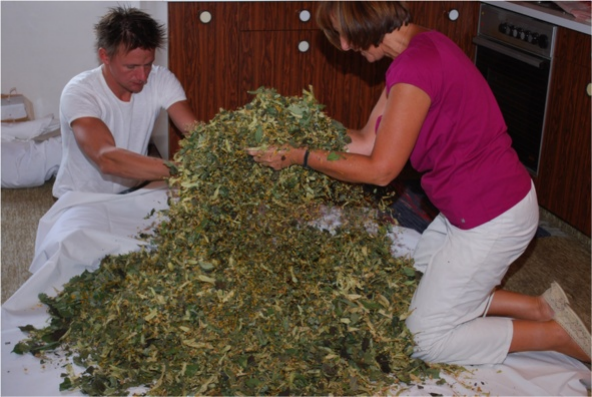
Blending of tea herbs for sale. Generating income is a rarely mentioned motivation for wild plant gathering (Photo: S. Grasser)

In the GWT a few local people gather wild plants for income. The most important marketed wild plant product is herbal tea (Fig. 7) [9]. Gathering for commercialisation is found in most parts of Europe and wild plants are gathered and marketed mainly for food and medical uses. Occasionally evergreens, mosses, twigs and leaves are marketed for decoration [23, 38, 39]. Wild plants are not a source of income for many people, although their market potential is promising, as confirmed by a recent study in Switzerland [39].

Gathering wild plants for income might involve less sustainable harvesting practices when compared with harvesting for fun. Also conflicts between non-commercial and commercial gatherers, as well as between different social, cultural, economic groups of people, might occur due to heterogeneous gathering practices [21].

We did not identify such dissent in the GWT, where most of the commercial gathering is done by the *Bergtee* project for selling herbal teas and thereby promoting wild gathered plants. The project aims at conserving and valuing traditional knowledge about wild plants and is not profit-oriented, although the gatherers receive remuneration. Gatherers who work for *Bergtee* follow informal guidelines for gathering and processing wild plants, which are drafted by experience and enforced by mutual trust [9]. The embeddedness of commercial wild plant gathering in local structures of social organization might have beneficial effects on the sustainability of commercial wild plant harvesting.

### Not gatherer-type

The main motivations for not gathering wild plants in the GWT are the lack of time and knowledge.

Changing lifestyles and the associated shortage of time are frequently mentioned reasons for the abandonment of traditional wild plant gathering in Europe. Furthermore, wild plant gathering can be time intensive, as shown for the harvest of wild asparagus in Spain [40].

More than one third of the interviewees mention that not knowing plants sufficiently is a reason for not gathering wild plants. This may relate to not knowing which parts of plants can be used for what purposes or generally lacking knowledge for properly identifying wild plants.

Casual but medially widely distributed reports about intoxications as a result of consumption of falsely identified wild plants might also contribute that people untrained in plant identification refrain from gathering. Besides that some intentionally gathered and consumed wild food plants may have detrimental health effects, such as those with rich amounts of bioactive compounds [41], toxic alkaloids [2] or with very high amounts of oxalic acid [29].

The material developed and published within the frame of the project as well as the project activities themselves intended to enhance knowledge and exchange about wild plants. This can help to increase the motivation for wild plant gathering of those people who do not gather because of a lack of knowledge. Furthermore workshops, herbal walks and ritual ceremonies about wild plants are offered in the GWT for rising awareness and increasing valuation and knowledge about wild plants [9].

## Conclusion

This study set out to identify the motivations for wild plant gathering, types of gatherers and variations of motivation and thereby contribute to explaining the resurgent popularity of wild plant gathering in Europe.

The most pronounced motivations for wild plant gathering in the Grosses Walsertal (GWT) are to obtain high quality products and enjoying the activity of plant gathering itself. Maintaining traditions is less important, and solely a very small number of people gather for income. In the last decades, the motivation changed from an external extrinsic motivation of gathering because of necessity towards an internalized extrinsic motivation of gathering for the product quality and the intrinsic motivation of gathering for the pleasure of the activity itself. This internalization of motivations supports the persistence of wild plant gathering, a positive self-perception of gatherers and good quality of engagement with wild plant gathering [12].

The validity of these results for other fieldsites should be tested. A generalization of findings across different user groups might result in wrong interpretation and management decisions [42] because of the diversity of user groups and wild plant species, gathering sites, motivations and interests involved in wild plant gathering [43].

From our findings we hypothesize that 1) quality is an important motivation for gathering wild plants, even more in un- or less disturbed areas than in more polluted environments, like cities; 2) fun is a motivation for wild plant gathering in areas where local people detach from the necessity of wild plant gathering and find new approaches through the internalization of the motivation; 3) tradition is a motivation in areas where gathering traditions exist; 4) gathering for income and selling wild plant products is beneficial for promoting wild plant gathering; 5) gathering wild plants can be enhanced by supporting dissemination and exchange of wild plant knowledge; 6) the motivations for homegardening and wild plant gathering are by tendency similar within a region.

Besides testing these hypotheses, we suggest that future research in ethnobotany should explore what plant quality means for local people and should make closer links between motivation for wild plant gathering and so far unexplored variables like gathering location, gathered plant species and gathered plant part to explore why specific plant parts are gathered but others not.

Investigating the motivations for ethnobotanical activities explains why such activities are taken up, continued or abandoned and may predict future developments. It can be deducted that creating and maintaining accessible and unpolluted environments will foster wild plant gathering because the main motivations for wild plant gathering, product quality and fun, are secured. Propagating the motivations of product quality and fun in other regions might increase interest for wild plant gathering.

## Additional file

Additional file 1: Table S1. Raw data on motivations and socio-demographic variables.
